# Microsatellite instability and sex-specific differences of survival in gastric cancer after neoadjuvant chemotherapy without and with taxane: An observational study in real world patients

**DOI:** 10.1007/s00432-023-04691-5

**Published:** 2023-03-31

**Authors:** Theresa Hiltner, Meike Kohlruss, Anna-Lina Herz, Sylvie Lorenzen, Alexander Novotny, Alexander Hapfelmeier, Moritz Jesinghaus, Julia Slotta-Huspenina, Leila Sisic, Matthias M. Gaida, Wilko Weichert, Katja Ott, Gisela Keller

**Affiliations:** 1 Institute of Pathology, TUM School of Medicine, Technical University of Munich, Trogerstr. 18, 81675 Munich, Germany; 2 III. Medizinische Klinik and Poliklinik, TUM School of Medicine, Technical University of Munich, Munich, Germany; 3 Department of Surgery, TUM School of Medicine, Technical University of Munich, Munich, Germany; 4 Institute of AI and Informatics in Medicine, School of Medicine, Technical University of Munich, Munich, Germany; 5 Institute of General Practice and Health Services Research, School of Medicine, Technical University of Munich, Munich, Germany; 6 Institute of Pathology, University of Marburg, Marburg, Germany; 7 Department of General, Visceral and Transplantation Surgery, University of Heidelberg, Heidelberg, Germany; 8 Institute of Pathology, University of Heidelberg, Heidelberg, Germany; 9 Institute of Pathology, University Medical Center Mainz, JGU-Mainz, Mainz, Germany; 10 TRON-Translational, Oncology at The University Medical Center of The Johannes Gutenberg University gGmbH, Mainz, Germany; 11 Institute of Pathology, German Cancer Consortium [DKTK], Partner Site Munich, Munich, Germany; 12 Department of Surgery, Klinikum Rosenheim, Rosenheim, Germany

**Keywords:** Adenocarcinoma, Gastric, Microsatellite instability, Sex, Prognosis, Neoadjuvant chemotherapy

## Abstract

**Purpose:**

To investigate the prognostic role of microsatellite instability (MSI) in association with sex of patients treated with platinum/fluoropyrimidine neoadjuvant chemotherapy (CTx) with or without a taxane-containing compound.

**Methods:**

Of the 505 retrospectively analyzed patients with gastric or gastroesophageal adenocarcinoma, 411 patients were treated without taxane and 94 patients with a taxane-containing compound. MSI was determined using standard assays.

**Results:**

Females demonstrated a better overall survival (OS) than males in the non-taxane group (HR, 0.59; 95% CI 0.41–0.86; p = 0.005), whereas no significant difference was found in the taxane group (HR 1.22; 95% CI 0.55–2.73, p = 0.630). MSI-High (-H) was associated with a better prognosis in both groups (without taxane: HR 0.56; 95% CI 0.33–0.97; p = 0.038; with taxane: HR 0.28; 95% CI 0.04–2.02, p = 0.204). In the non-taxane group, female MSI-H patients showed the best OS (HR 0.18, 95% CI 0.05–0.73; p = 0.016), followed by the female microsatellite stable (MSS) (HR 0.67, 95% CI 0.46–0.98, p = 0.040) and the male MSI-H group (HR 0.76; 95% CI 0.42–1.37, p = 0.760) taken the male MSS group as reference. In the taxane group, female and male MSI-H patients demonstrated the best OS (female MSI-H: HR 0.05, 95% CI 0.00–240.46; male MSI-H: HR 0.45, 95% CI 0.61–3.63, p = 0.438), whereas the female MSS group showed a decreased OS (HR 1.39 95% CI 0.62–3.12, p = 0.420) compared to male MSS patients.

**Conclusion:**

OS in gastric/gastroesophageal cancer after CTx might depend on sex and MSI status and may differ between patients treated with or without a taxane compound in the chemotherapeutic regimen.

**Supplementary Information:**

The online version contains supplementary material available at 10.1007/s00432-023-04691-5.

## Introduction

Microsatellite instability (MSI) is characterized by the accumulation of length alterations in short, repetitive DNA sequences. These alterations occur because of errors during DNA replication and indicate defects in the DNA mismatch repair system, which is normally responsible for correcting these mistakes. A high degree of MSI (MSI-H) is found in 10–20% of gastric carcinomas, and The Cancer Genome Atlas project (TCGA) identified MSI-H tumors as a distinct molecular tumor class (Cancer Genome Atlas Research 2014, Polom et al. 2018; Kohlruss et al. 2019). An association between MSI-H and specific clinicopathological features, such as female sex, antral tumor localization, intestinal histology, and better prognosis, has been described in several studies (Cancer Genome Atlas Research 2014, Polom et al. 2018; Kohlruss et al. 2019). Recently, MSI-H has gained considerable attention as a predictive biomarker for the application of immune checkpoint therapy, and MSI status was found to be a predictive biomarker for pembrolizumab therapy in advanced gastric or gastroesophageal junction cancer (Le et al. 2015; Chao et al. 2021). In contrast, the predictive role of MSI in the setting of neoadjuvant chemotherapy for these tumors is controversially discussed (Lordick 2020; Smyth 2020). MSI has been reported as a biomarker indicating not only no response to CTx, but also a detrimental effect in MSI patients treated with epirubicin, cisplatin, and 5FU compared to patients treated with surgery alone in the frame of the MAGIC trial (Smyth et al. 2017; Pietrantonio et al. 2019). However, an association between MSI and a good prognosis after neoadjuvant CTx or immunotherapy has been demonstrated by others (Haag et al. 2019; Kohlruss et al. 2019; Andre et al. 2023).

In a recent study, we added a further aspect to this issue, as we showed that sex may be related to patient outcomes. In particular, females with MSI-H tumors showed extraordinarily good survival after neoadjuvant CTx compared to males with MSI-H and microsatellite stable (MSS) tumors, and also compared to females with MSS tumors (Kohlruss et al. 2021). Similar results were demonstrated in a large meta-analysis including 1307 tumors (Quaas et al. 2022).

Regarding optimized therapeutic modalities for gastric and gastroesophageal cancer patients, a significant increase in survival was shown for patients treated with inclusion of the taxane compound docetaxel together with 5FU, leucovorin, and oxaliplatin in the FLOT 4 trial (Al-Batran et al. 2019). Since then, this protocol has been recommended as the standard of care for patients who can tolerate it (Lordick et al. 2022).

In our previous study, the majority of patients were treated with fluoropyrimidine/platinum-based neoadjuvant CTx without taxanes. Hence, the question arises whether the prognostic role of MSI and sex may be similar or different in the two populations of patients treated with chemo-therapeutic protocols, including or excluding taxane. Thus, we enlarged our patient cohort by including mainly patients treated with the FLOT regimen. The goal of the present study was to investigate the role of MSI and sex in two relatively homogeneous patient cohorts stratified according to the addition of a taxane compound to the therapeutic regimen.

## Patients and methods

### Patients and chemotherapy

Tumors from patients with gastric adenocarcinomas (GC), including tumors of the gastro-esophageal junction (GEJ) (Siewert type II and III) (Siewert et al. 1998) that were treated with neoadjuvant CTx between 2004 and 2012 at the Department of Surgery of the University of Heidelberg and between 1994 and 2018 at the Technical University of Munich were included in the study. Patients were treated with a platinum/fluoropyrimidine neoadjuvant CTx without or with taxane. The chemotherapy regimens are listed in detail in Supplementary Table S1.

The inclusion criteria for the present study were treatment with the specified chemotherapy and the availability of DNA or paraffin blocks from tumor and nontumorous tissues. In total, 394 patients were included in our previous study (Kohlruss et al. 2021).

### Response evaluation

The response to neoadjuvant CTx was evaluated as previously described (Becker et al. 2011). In brief, tumor regression was determined histopathologically and classified into three tumor regression grades (TRG): TRG1a/b, TRG2, and TRG3, which corresponded to complete/ < 10%, 10–50% and > 50% residual tumor cells within the tumor bed, respectively. Patients with TRG1 were classified as responders, whereas those with TRG2 and TRG3 were classified as non-responders.

### Follow-up and overall survival

The follow-up was performed as previously described (Kohlruss et al. 2019). The median follow-up period for the patients treated with neoadjuvant CTx without taxane was 60.7 months (interquartile range [IQR] 35.3–82.2 months) and for patients treated with inclusion of taxane, the median was 37.3 months (IQR, 20.6–63.8 months). Overall survival (OS) was defined as the time between the date of surgery and death from any cause.

### Ethics statement

The study was conducted in accordance with the Declaration of Helsinki and was approved by the local Institutional Review Boards at the Technical University of Munich (reference: 502/15 s) and University of Heidelberg (reference: 301/2001).

### Molecular analysis

Molecular analyses, including DNA isolation, analysis, and definition, were described in detail in our previous study (Kohlruss et al. 2021). DNA from normal and tumor tissues was isolated from formalin-fixed paraffin-embedded (FFPE) tissues after manual microdissection. MSI was analyzed using five markers encompassing two mononucleotide repeat markers, BAT25 and BAT26, and three dinucleotide repeat markers D2S123, D5S346, and D17S250, as recommended by the National Cancer Institute (Boland et al. 1998). According to a standardized definition, MSI-H was defined if at least two of the five markers showed MSI and as low (L) MSI if only one of the five markers showed MSI. To avoid classification of MSI-H based on tumors with instabilities at only two dinucleotide markers, the tumors were additionally analyzed using three mononucleotide markers: NR-21, NR24, and NR-27. As described in detail in our previous study, those with no instability at these mononucleotide repeats were classified as MSI-L (Kohlruss et al. 2021). If no instabilities were observed, tumors were classified as microsatellite stable (MSS).

### Statistical analysis

Chi-squared tests or Fisher's exact tests were used for hypothesis testing of the differences between relative frequencies. The Mann–Whitney *U* test was used to compare continuously scaled variables. Kaplan–Meier estimates of survival probabilities were compared using the log-rank test. Relative risks were estimated using hazard ratios (HRs) with 95% confidence intervals (95% CI) from univariable and multivariable Cox proportional hazards models. Multivariable models were built by stepwise forward variable selection using Wald tests to control for model complexity and the respective number of included prognostic factors, given the limited number of observed events. The interaction effects of MSI and sex were included in the model formulas to explore the sex-specific effects of MSI. All statistical analyses were performed using SPSS, Version 25 (IBM Corp., Armonk, NY, USA). Exploratory 5% significance levels (two-tailed) were used for the hypothesis testing.

## Results

### Patient characteristics treated with platinum/fluoropyrimidine neoadjuvant CTx without or with taxane

Tumors from 505 patients were included in the study. Tumor biopsies before CTx were available from 253 patients and resected specimens after CTx from 252 patients. For 59 of the 505 patients, corresponding pretherapeutic biopsies before CTx and resected specimens after CTx were available for analysis, and concordant results regarding MSI-H were found in each case. Thus, for the purpose of this study, patients with biopsies before CTx and resected specimens after CTx were combined and analyzed as described previously (Kohlruss et al. 2021).

**Fig. 1:**
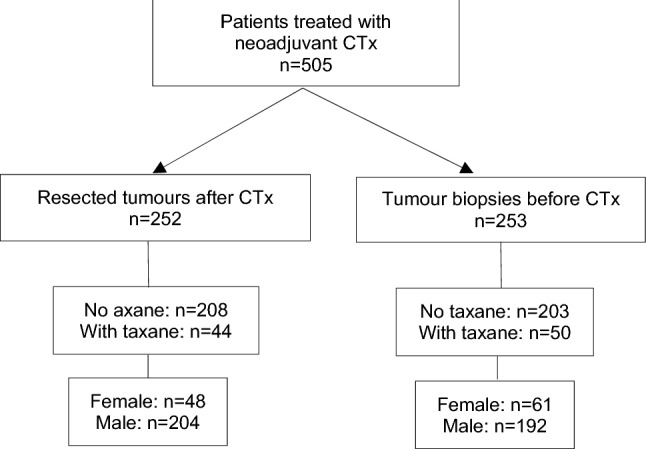
Overview of the study

Of the 505 patients, 411 were treated with platinum/fluoropyrimidine-based CTx without a taxane and 94 patients with a taxane-containing compound (Supplementary Table S1). An overview of the study population, including the numbers of females and males is shown in Fig. 1. The patient characteristics are shown in Table 1.

**Table 1:** Patient characteristics

Category	Value	All patients *n* (%)	CTx without taxane *n* (%)	CTx with taxane *n* (%)	*P* value^a^
Cases	Total	505 (100)	411 (100)	94 (100)	
Age [yr]	Median	60.9	62.0	56.2	< 0.001
	Range	23.3–81.3	23.3 -81.3	29.2–76.7	
Sex	Male	396 (78.4)	322 (78.3)	74 (78.7)	0.936
	Female	109 (21.6)	89 (21.7)	20 (21.3)	
Tumor localization	Proximal	318 (63.0)	261 (63.5)	57 (60.6)	0.604
	Non-proximal	187 (37.0)	150 (36.5)	37 (39.4)	
Laurén classification	Intestinal	286 (56.6)	234 (56.9)	52 (55.3)	0.776
	Non-intestinal	219 (43.4)	177 (43.1)	42 (44.7)	
Clinical tumor stage (cT)	T2	26 (5.2)	20 (5.0)	6 (6.5)	0.561
	T3/4	470 (94.8)	383 (95.0)	87 (93.5)	
	n/a	9	8	1	
Clinical lymph node status (cN)	Negative Positive n/a	76 (16.9) 374 (83.1) 55	59 (16.6) 297 (83.4) 55	17 (18.1) 77 (81.9)	0.775
ypT^b^	0,1,2	115 (22.9)	96 (23.5)	19 (20.2)	0.214
	3,4	388 (77.1)	313 (76.5)	75 (79.8)	
	n/a	2	2		
ypN^b^	Negative	169 (33.6)	130 (31.8)	39 (41.5)	0.072
	Positive	334 (66.4)	279 (68.2)	55 (58.5)	
	n/a	2	2		
Resection category	R0	380 (75.5)	307 (75.1)	73 (77.7)	0.597
	R1	123 (24.5)	102 (24.9)	21 (23.3)	
	n/a	2	2		
Tumor regression grade (TRG)	1a 1b 2 3	13 (2.6) 54 (10.7) 188 (37.2) 250 (49.5)	11 (2.7) 42 (10.2) 150 (36.5) 208 (50.6)	2 (2.1) 12 (12.8) 38 (40.4) 42 (44.7)	0.724
Responder Nonresponder	TRG1a/b TRG2, 3	67 (13.3) 438 (86.7)	53 (12.9) 358 (87.1)	14 (14.9) 80 (85.1)	0.606
EBV status	positive negative n/a	23 (4.6) 481 (95.4) 1	18 (4.4) 393 (96.6)	5 (5.4) 88 (94.6) 1	0.782
MSI status	MSS MSI-L MSI-H	428 (84.8) 29 (5.7) 48 (9.5)	350 (85.2) 23 (5.6) 38 (9.2)	78 (83.0) 6 (6.4) 10 (10.6)	0.705
MSI status	MSS/MSI-L MSI-H	457 (90.5) 48 (9.5)	373 (90.8) 38 (9.2)	84 (89.4) 10 (10.6)	0.678

*CI* confidence interval, n/a not available

^a^Chi square or Fisher test; for age: Mann–Whitney *U* test

^b^TNM and UICC classification according to 7th Edition of UICC

Patients in the non-taxane group were significantly older compared to the patients treated with inclusion of taxane (median age 62.0 compared to 56.2, p < 0.001). A somewhat higher frequency of patients with positive lymph nodes (p = 0.072) was observed in the non-taxane group. Other patient characteristics showed no obvious differences, specifically regarding sex; the frequencies of 78.3 and 78.7% of male patients in the non-taxane and taxane groups, respectively, were nearly the same.

In addition, we compared the sex-specific patient characteristics in the non-taxane and the taxane group. Details are shown in Table 2. In both treatment groups, there was a significant association of male sex with proximal tumor localization (non-taxane group: p < 0.001; taxane group: p = 0.041) and intestinal histotype (non-taxane group: p = 0.005; taxane group: p = 0.046). A significant higher frequency of lower tumor stages after CTx was observed for females in the non-taxane group (p = 0.016). No other statistically significant differences were observed.

**Table 2:** Sex-specific characteristics of patients treated without and with taxane

Category	Value	CTx without taxane			CTx with taxane	
					Female* n* (%)	Male* n* (%)
		Female* n* (%)		Male* n* (%)		
Cases	Total	89 (100)		322 (100)	20 (100)	74 (100)
Age [yr]	Median	62.3		62.0	57.7	55.85
	Range	28.3 – 79.2		23.3–81.3	31.9 – 75.2	29.3 – 76.7
Tumor localization	Proximal^b^	42 (47.2)		219 (68.0)	8 (40.0)	49 (66.2)
	Non-proximal	47 (52.8)		103 (32.0)	12 (60.0)	25 (33.8)
		p^a^ < 0.001			p^a^ = 0.041	
Laurén classification	Intestinal	39 (43.8.)		195 (60.6)	7 (35.0)	45 (60.8)
	Non-intestinal	50 (56.2)		127 (39.4)	13 (65.0)	29 (39.2)
		p^a^ = 0.005			p^a^ = 0.046	
Clinical tumor stage (cT)	T2	6 (7.1)		14 (4.4)	2 (10.0)	4 (5.5)
	T3/4	79 (92.9)		304 (95.6)	18 (90.0)	69 (94.5)
	n/a	4		4		1
Clinical	negative	15 (19.5)		44 (15.8)	4 (20.0)	13 (17.6)
lymph node	positive	62 (80.5)		235 (84.2)	16 (80.0)	61 (78.4)
status (cN)	n/a	12		43		
ypT^c^	0,1,2	29 (33.3)		67 (20.8)	5 (25.0)	14 (18.9)
	3,4	58 (66.7)		255 (79.2)	15 (75.0)	60 (81.9)
	n/a	2				
		p^a^ = 0.016				
ypN^c^	Negative	32 (36.8)	98 (30.4)		11 (55.0)	28 (37.8)
	Positive	55 (63.29	224 (69.6)		9 (45.0)	46 (62.2)
	n/a	2				
Resection category	R0	69 (79.3)	238 (73.9)		17 (85.0)	56 (75.7)
	R1	18 (20.7)	84 (26.1)		3 (15.0)	18 (24.3)
	n/a					
Tumor regression grade (TRG)	1a 1b 2 3	6 (6.7) 8 (9.0) 30 (33.7) 45 (50.6)	5 (1.6) 34 (10.6) 120 (37.3) 163 (50.6)		1 (5.0) 4 (20.0) 5 (25.0) 10 (50.0)	1 (1.4) 8 (10.8) 33 (44.6) 32 (43.2)
Responder Nonresponder	TRG1a/b TRG2, 3	14 (15.7) 75 (84.3)	39 (12.1) 283 (87.9)		5 (25.0) 15 (75.0)	9 (12.2) 65 (87.8)
EBV status	Positive Negative n/a	1 (1.1) 88 (98.9)	17 (5.3) 305 (94.7)		0 (0) 20 (100)	5 (6.8) 68 (93.2) 1
MSI status	MSS MSI-L MSI-H	72 (80.9) 5 (5.6) 12 (13.5)	278 (86.3) 18 (5.6) 26 (8.1)		16 (80.0) 1 (5.0) 3 (15.0)	62 (83.8) 5 (6.8) 7 (9.5)
MSI status	MSS/MSI-L MSI-H	77 (86.5) 12 (13.5)	296 (91.9) 26 (8.1)		17 (85.0) 3 (15.0)	67 (90.5) 7 (9.5)

Only significant p –values < 0.05 were included in the table. CI, confidence interval; n/a, not available

^a^Chi square test

^b^gastroesophageal junction tumors according to Siewert type II and III

^c^TNM and UICC classification according to 7th Edition of UICC

### Response and survival of the patients treated with platinum/fluoropyrimidine neoadjuvant CTx without or with taxane

Patients treated without a taxane containing regimen showed a decreased survival (hazard ratio [HR]: 1.27, 95% confidence interval CI 0.90–1.82, p = 0.179) (Supplementary Fig. S1). The median OS in the non-taxane group was 38.7 months (95%CI: 25.14–52.27) and 67.7 months (95% CI 39.04–96.36) in the taxane group.

In the non-taxane group, 12.9% of patients were histopathological responders (TRG1) (Table 1). Responding patients showed a significantly better OS than non-responding patients (TRG2/TRG3) in this group (HR: 0.32, 95% CI 0.19–0.56, p < 0.001) (Supplementary Fig. S2A). The median OS was not reached in the responding and was 31.1 months (95% CI: 23.6–38.6) in the non-responding group.

In the taxane group, 14.9% of the patients responded (Table 1). An obviously better OS of responding compared to non-responding patients was also observed in this group (HR: 0.31, 95% CI 0.77–1.23, p = 0.105) (Supplementary Fig. S2B). The median OS for the responding patients was not reached and was 55.7 months (95% CI 22.0–89.4) for the non-responding patients.

### Sex and survival of patients treated with platinum/fluoropyrimidine neoadjuvant CTx without or with taxane

**Fig. 2:**
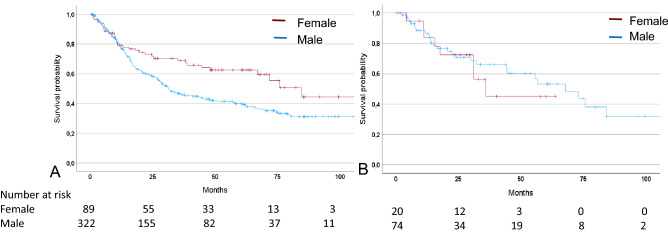
Discrimination of patients’ survival by sex. Kaplan–Meier curves of female and male patients are shown. Patients treated with neoadjuvant CTx **A** without taxane, p = 0.005; **B** with taxane, p = 0.630; p of univariable Cox-regression

In the non-taxane group, females demonstrated a significantly increased OS (HR: 0.59, 95% CI 0.41–0.86, p = 0.005) compared to men. The median OS was 84.4 months (95% CI 63.8 – 105.0) for women and 31.3 months (95% CI 23.7–39.0) for men (Fig. 2A).

In the taxane group, a decreased OS was seen in females; however, this was not statistically significant (HR 1.22, 95% CI 0.55–2.73, p = 0.630). The median OS was 35.9 months (95% CI, not given) for women compared to 67.7 months (95% CI 46.0–89.4) for men (Fig. 2 B).

Survival data are summarized in Table 3.

**Table 3:** Survival data of patients treated with neoadjuvant CTx without and with taxane and sex

CTx	Sex	No	Events	Median OS [mo] (95% CI)	HR (95% CI)	p^a^-value
Without taxane	Female	89	34	84.4 (63.8–105.0)	0.59 (0.41–0.86)	0.005
	Male	322	177	31.3 (23.7–39.0)	1 ref	
With taxane	Female	20	8	35.9 (−)	1.22 (0.55–2.73)	0.630
	Male	74	28	67.7 (46.0–89.4)	1 ref	

CI confidence interval, HR Hazard ratio, ref reference nr not reached, ^a^univariable Cox-regression

### Frequency of MSI and association with patients characteristics

In the non-taxane group, 38 of 411 (9.2%) tumors showed MSI-H, and 373 (90.8%) were classified as MSS/MSI-L (Table 1). The MSI-H was significantly associated with older age in this group (p = 0.044).

In the taxane group, 10 of 94 (10.6%) tumors showed MSI-H, and 84 (89.4%) were classified as MSS/MSI-L (Table 1). There was a significant association between MSI status and tumor localization in this group, as eight of 37 (21%) non-proximal and two of 57 (3.5%) proximal (GEJ Siewert type II and III) tumors showed MSI-H (p = 0.008).

No statistically significant associations with the response to CTx or other clinical characteristics were found in either group. The results are summarized in Supplementary Table S2.

### MSI and survival of patients treated with platinum/fluoropyrimidine neoadjuvant CTx without or with taxane

**Fig. 3:**
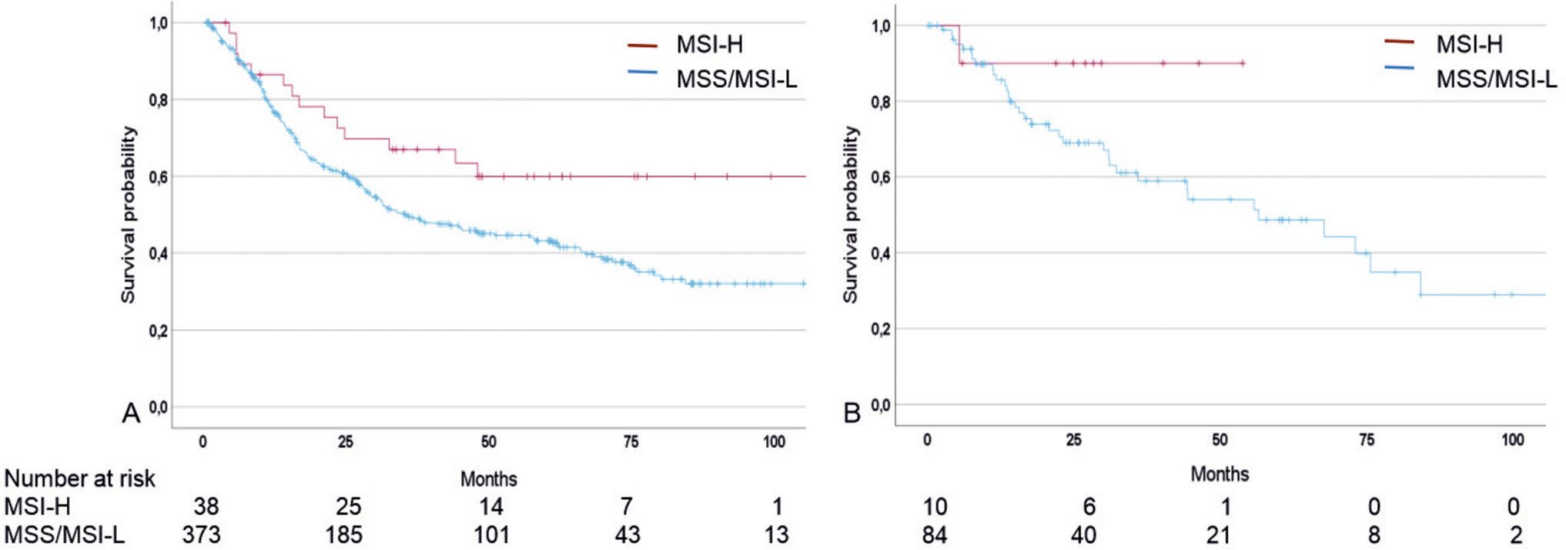
Discrimination of patients’ survival by MSI status, Kaplan–Meier curves of patients with MSI-H and MSS/MSI-L tumors are shown. Patients treated with neoadjuvant CTx **A** without taxane, p = 0.038; **B** with taxane, p = 0.204; p of univariable Cox-regression

In the non-taxane group, MSI-H was significantly associated with increased OS compared to patients with MSS/MSI-L tumors (HR: 0.56; 95% CI 0.33–0.97, p = 0.038). The median OS was not reached for MSI-H patients and was 35.60 months (95% CI 24.1–47.1) in the MSS/MSI-L group (Fig. 3A).

In the taxane group, patients with MSI-H tumors also showed a clearly prolonged survival; however, probably due to the small number of tumors, the difference was not statistically significant (HR: 0.28, 95% CI 0.04–2.02, p = 0.204). The median OS of MSI-H patients was not reached and was 56.5 months (95% CI 30.3–82.7) for patients with MSS/MSI-L tumors (Fig. 3B). Survival data are summarized in Table 4.

**Table 4:** Survival data of patients treated with neoadjuvant CTx without and with taxane and MSI status

CTx	MSI status	No	Events	Median OS [mo] (95% CI)	HR (95% CI)	p^a^-value
Without Taxane	MSI-H	38	14	n.r	0.56 (0.33–0.97	0.038
	MSS/MSI-L	373	197	35.6 (24.1–47.1)	1 ref	
With Taxane	MSI-H	10	1	n.r	0.28 (0.04–2.02)	0.204
	MSS/MSI-L	84	35	56.5 (30.3–82.7)	1 ref	

*CI* confidence interval, *HR* Hazard ratio *ref*., reference, *nr* not reached; ^a^univariable Cox-regression

### Sex-specific MSI status and survival of patients treated with platinum/fluoropyrimidine neoadjuvant CTx with or without taxane

**Fig. 4:**
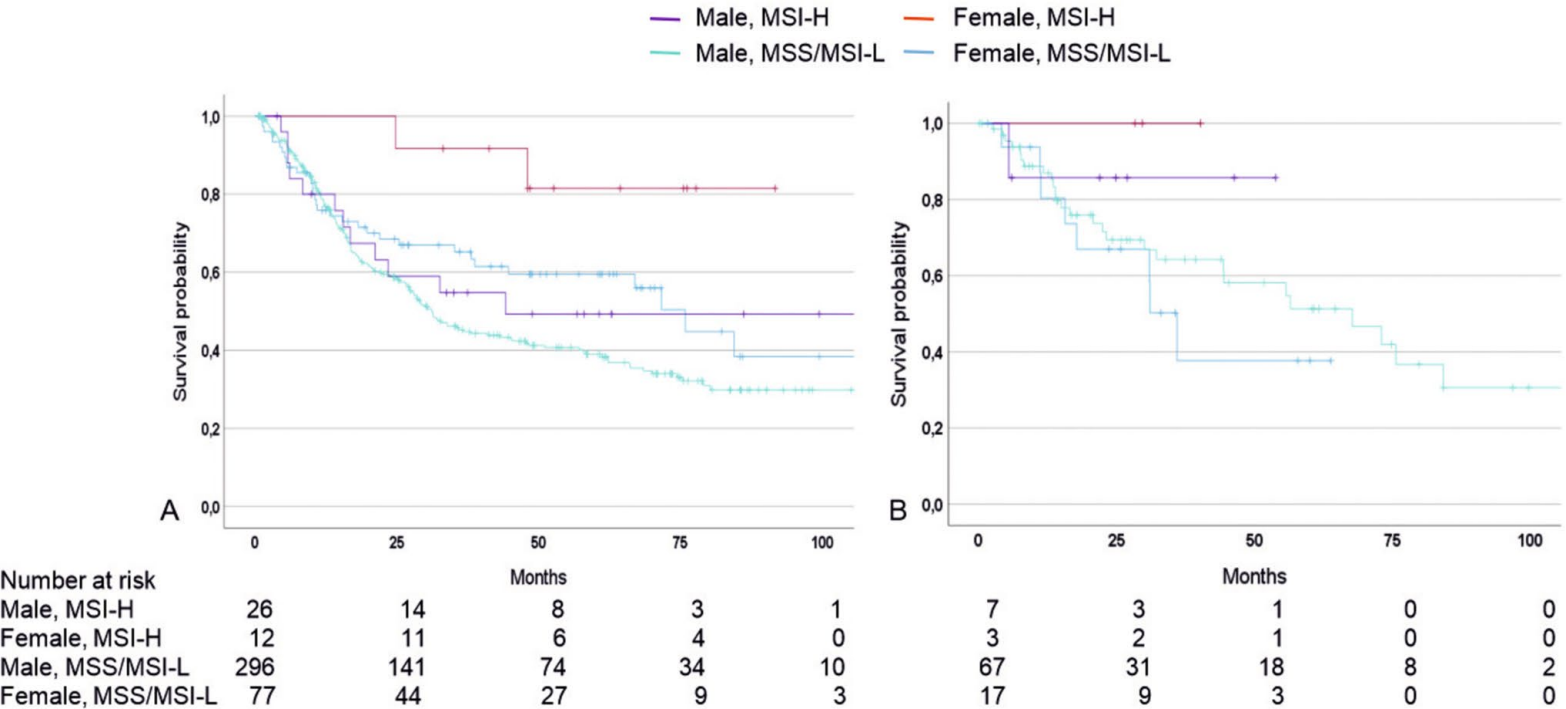
Discrimination of patients’ survival by MSI status and sex**,** Kaplan–Meier curves of female and male patients with MSI-H and MSS/MSI-L tumors are shown. Patients treated with neoadjuvant CTx **A** without taxane, p = 0.019; **B** with taxane, p = 0.706; Cox-regression overall p of group comparison

Considering OS in the sex-specific MSI subgroups and taken the male MSS/MSI-L group (n = 296/411) as a reference, female MSI-H patients (n = 12/411) showed the best OS (HR: 0.18; 95% CI 0.05–0.73, p = 0.016), followed by the female MSS (n = 77/411) (HR: 0.67, 95% CI 0.46–0.98, p = 0.040) and then the male MSI-H group (n = 26/411) (HR: 0.76, 95% CI 0.42–1.37, p = 0.760) in the non-taxane group (Fig. 4A).

In the taxane group, women also demonstrated the best OS (HR: 0.05, 95% CI 0.00–240.46). As there were only three females among the ten MSI-H patients, no p-value is given for this specific subgroup. The male MSI-H patients (n = 7/94), however, also showed an obviously better survival with a clearly reduced risk (HR: 0.45, 95% CI 0.61–3.63, p = 0.438) compared to the male MSS/MSI-L patients (n = 67/94) as a reference, whereas the female MSS/MSI-L group (n = 17/94) showed an increased risk (HR: 1.39, 95% CI 0.62–3.12, p = 0.420) (Fig. 4B).

Detailed survival data are summarized in Table 5.

**Table 5:** Survival data of patients treated with neoadjuvant CTx without and with taxane and MSI status in association with sex

	Sex and MSI status	No	Events	Median OS [mo] (95% CI)	HR (95% CI)	p-value^a^	p-value^b^
Without Taxane	Female, MSI-H	12	2	n.r	0.181 (0.05–0.73)	0.016	0.019
	Female, MSS/MSI-L	77	32	75.8 (62.0–89.6)	0.67 (0.46–0.98)	0.040	
	Male, MSI-H	26	12	44.1	0.76 (0.42–1.37)	0.760	
	Male, MSS/MSI-L	296	165	31.1 (25.6–36.6)	1 ref	–	
With Taxane	Female, MSI-H	3	0	n.r	0.05 (0.0–240.46)	0.479	0.706
	Female, MSS/MSI-L	17	8	35.9 (28.9–42.9)	1.39 (0.62–3.12)	0.420	
	Male, MSI-H	7	1	n.r	0.45 (0.61–3.63)	0.438	
	Male, MSS/MSI-L	67	27	67.7 (15.0–38.3)	1 ref	–	

*CI* confidence interval, *HR* Hazard ratio, *ref*. reference, *nr* not reached

^a^Cox regression p against reference

^b^Cox regression overall p of group comparison

### Multivariable analysis

Multivariable analysis of all patients adjusted for pre-therapeutically available variables (age, cT, tumor localization, histological type, CTx without and with taxane) that were included by variable selection and including the interaction of MSI status and sex as factors revealed age (p = 0.018), cT (p = 0.016), and the interaction of MSI status and sex (p = 0.007) as significant prognostic factors.

Multivariable analysis adjusting for the post-therapeutically available factors (age, ypT, ypN, and R-category, tumor localization, histological type, CTx with and without taxane, response to CTx) that are included by variable selection and the interaction of MSI status and sex revealed that age (p = 0.006), ypT, ypN, and R status (each p < 0.001), and the interaction of MSI and sex (p = 0.016) were significant prognostic factors. The results are summarized in Table 6.

**Table 6:** Multivariable model of survival adjusted for pre-and post-therapeutically available clinical factors, including the interaction of MSI status and sex

All patients n = 505	HR	95% CI	p Value^a^
Pre-therapeutic factors^b^			
Age	1.02	1.00–1.03	0.018
Clinical tumor stage			
cT2	1 ref	–	0.016
cT3/4	2.52	1.19–5.36	
Interaction MSI status and sex	0.15	0.04–0.59	0.007
Post-therapeutic factors^c^			
Age	1.02	1.01–1.03	0.006
ypT^d^			< 0.001
(y)pT0	1 ref	–	
(y)pT1	1.36	0.14–13.10	0.791
(y)pT2	5.03	0.68–37.10	0.113
(y)pT3	5.30	0.74–38.06	0.097
(y)pT4	9.72	1.34–70.66	0.025
ypN			
pN0	1 ref	–	< 0.001
pN positiv	2.77	1.97–3.91	
R status			
R0	1 ref	–	< 0.001
R1	1.94	1.46–2.57	
Interaction MSI-status and sex	0.18	0.04–0.72	0.016

*ref* reference, *MSS/MSI-L* microsatellite stable/low microsatellite instability, *MSI-H* high microsatellite instability, *HR* Hazard ratio, *CI* confidence interval

^a^Wald-Test of Hazard Ratio

^b^Pre-therapeutic factors included sex, age, localization (proximal vs. non-proximal), Laurén subtypes (intestinal vs. non-intestinal), neoadjuvant chemotherapy (without vs. with taxane), clinical tumor stage (cT2 vs. cT3/4), and MSI status (MSS/MSI-l vs. MSI-H)

^c^Post-therapeutic factors included: sex, age, localization (proximal vs. non-proximal), Laurén subtypes (intestinal vs. non-intestinal), neoadjuvant chemotherapy (without vs. with taxane), ypT (pT1-pT4), ypN, R-category, Response (TRG1 vs. TRG2,3), MSI status (MSS/MSI-L vs. MSI-H)

^d^TNM classification according to 7th Edition UICC

## Discussion

There is growing evidence that sex-specific differences in tumor biology may influence the survival and outcome of specific chemo- and immune-based therapies in cancer patients, and the relevance of investigating disaggregated data by sex in research has been emphasized recently (Wagner et al. 2019, 2022, Ozdemir et al. 2022). In our previous study, we showed that women with MSI-H tumors, in particular, showed superior survival after neoadjuvant CTx. Notably, the majority of patients in that study were treated with platinum/fluoropyrimide-based CTx, not including a taxane-containing compound (Kohlruss et al. 2021).

Since the report of significantly better survival of patients treated with 5FU, leucovorin, oxaliplatin, and docetaxel (FLOT), the FLOT regimen is considered the standard approach for gastric cancer patients (Al-Batran et al. 2019; Lordick et al. 2022). In the present study, we enlarged our patient cohorts mainly by including platinum/fluoropyrimidine-based patients treated with taxane and evaluated the cohorts treated with and without a taxane compound separately for sex-specific differences regarding prognosis of the patients with a specific focus on the MSI status of the tumors.

As shown in our previous study and confirmed by others, this retrospective exploratory analysis confirmed that patients with MSI-H tumors demonstrated significantly increased survival after neoadjuvant CTx (Kohlruss et al. 2021; Quaas et al. 2022). This superior survival was specifically conspicuous considering the striking good survival of females, but not of men with MSI-H tumors in the group of patients treated without taxane. This finding may be related to stronger immune reactions of women with an already immunogenic “hot” MSI-H tumor and an additional boosting effect of CTx (Klein et al. 2016, Rivera Vargas et al. 2017, Bruni et al. 2020; Kohlruss et al. 2021). In the group of patients treated with the addition of a taxane-containing compound, patients with MSI-H tumors also demonstrated considerably better survival than patients with microsatellite stable tumors. Similar to our results, an Italian group demonstrated that MSI-H was a positive prognostic marker in perioperatively FLOT-treated patients (Giommoni et al. 2021). In addition, in the multicenter, retrospective PROSECCO study, the outcome of patients with MSI-H was remarkably better than that of patients with MSS after treatment according to the FLOT protocol (Nappo et al. 2023). Furthermore, preliminary results of the DANTE study analyzing FLOT in comparison with FLOT and atezolizumab showed high rates of pathological tumor regression in MSI patients, with the current restriction that survival data are not yet available, and translation of pathological complete response into improved survival of the patients has to be shown (Al-Batran et al. 2022). Notably, mismatch repair deficient patients treated with FLOT alone showed an improved pathological complete response rate of 27% (Al-Batran et al. 2022), disproving the hypothesis of retrospective analysis that chemotherapy in locally advanced MSI-H patients has a detrimental effect (Smyth et al. 2017). Thus, these findings are in line with our results.

Interestingly, considering the sex-specific differences in the MSI-H patients in the taxane group in our study, a marked increase in survival was also observed in male patients with MSI-H. We are aware that due to the small number of patients, our findings must be interpreted with caution, and further analysis in enlarged patient cohorts should be performed. However, it is tempting to speculate and delivers first evidence that taxane treatment may improve the prognosis of men with MSI-H tumors. Moreover, this may also apply to men with microsatellite stable tumors, as a survival advantage of females seen in the treatment group without taxane was not observed in the taxane group.

Sex differences in taxane toxicities have been described, and they are related to several biological factors, such as unequal pharmacokinetics and varied adverse drug reactions, which are higher in females than in men. Taxanes are known to act on androgen receptor signaling, inducing microtubule dysfunction, which is considered a main target of taxanes exerting their antineoplastic effect (Chmielewski and Limoli 2022). In addition, as outlined in a comprehensive review by Chmielewski (Chmielewski and Limoli 2022), several studies in animal and cell line models indicate, that estrogen and androgens may have opposite roles in microtubule polymerization, leading to male-specific susceptibility and female-specific protection regarding the effect of taxane (Kipp et al. 2003, Kampa et al. 2006; Chmielewski and Limoli 2022).

In respect to baseline patient characteristics, sex-specific associations of tumor localization and histotype were observed. However, we found similar proportions in both treatment groups. Therefore, a relationship of these associations to sex-specific survival differences in only one of the treatment groups seems unlikely. In addition, a large meta-analysis of four randomized clinical trials, in which all of them did not include a taxane containing chemotherapeutic agent (Athauda et al. 2020), is in line with our finding of a general better outcome of females after CTx without taxane. Thus, the outcome of patients after neoadjuvant CTx may be influenced by sex and MSI, which may differ depending on the particular multimodal treatment applied. Our findings shed some light on the apparently controversial discussion regarding the most appropriate CTx treatment for MSI-H patients (Lordick 2020; Smyth 2020). This was initiated by analyzing resected specimens of the patients participating in the MAGIC trial, which was the first trial demonstrating the advantages of pre/perioperative CTx including epirubicin, cisplatin and 5FU compared to treatment with surgery alone in 2006 and since then has been the standard of care in Western countries for years (Cunningham et al. 2006; Lordick et al. 2022). The results regarding the prognostic relevance of MSI-H in this trial and in subsequent reports indicated that pre-/ perioperative CTx would not be beneficial to patients with MSI and may even harm them compared to patients treated with surgery alone (Smyth et al. 2017; Pietrantonio et al. 2019; Stolze et al. 2022). No sex-specific data of MSI-H patients are available, but owing to the relatively strong dominance of males in the study, the majority of MSI-H patients may be men (Cunningham et al. 2006; Smyth et al. 2017). In addition, the analysis of only resected specimens after preoperative CTx, which necessarily leads to the exclusion of responding patients with minimal or no residual tumor cells in the resected specimen, may introduce some bias and represent a limitation for generalization (Smyth et al. 2017, Stolze, Franke et al. 2022). Furthermore, several studies have not found a negative prognostic effect of MSI-H in the neoadjuvant chemotherapeutic setting (Haag et al. 2019; Hashimoto et al. 2019; Kohlruss et al. 2019; Quaas et al. 2021). It will be interesting to see the results of ongoing clinical trials evaluating chemotherapy in combination with an immune checkpoint inhibitor, as in the MATTERHORN and KEYNOTE-585 trials (Bang et al. 2019; Janjigian et al. 2022).

Taken together, we believe that a general recommendation not to treat MSI-H patients with neoadjuvant CTx is not justified, as female MSI-H patients show a strikingly good survival after CTx with a conventional treatment regimen without taxane. In addition, there is increasing evidence that MSI-H patients, regardless of sex, have an increased prognosis in therapeutic settings, including taxane-containing agents.

The limitations of our study, as already mentioned, refer mainly to its retrospective nature and the small number of taxane-treated patients, specifically when performing analyses within subgroups. A formal subgroup analysis including a direct comparison of effects between taxane-treated and non-taxane-treated patients with broad adjustment for potential confounding is outstanding. Thus, we emphasize the exploratory nature of our study, which may be considered as hypothesis generating and caution is necessary for the final interpretation of our data. In addition, as only half of our analyzed samples were tumor biopsies, we cannot exclude some bias due to a lack of major/complete responding patients. Response rates from patients treated at our Institutions in the range from 23 to 30% have been reported (Becker et al. 2011; Lorenzen et al. 2012; Springfeld et al. 2015). Thus inferring from this, 17% of patients with TRG1 were not included in the present study.

Another limitation is that our patients were not treated in the frame of a clinical trial, and the results represent “real-world” data. Although controlled randomized clinical trials are considered the gold standard to define the efficacy of treatment and are most suitable for finding relevant biomarkers, there is an increasing trend to emphasize and appreciate the opportunities and benefits of data arising from real-world evidence (Di Maio et al. 2020; Nazha et al. 2021).

In conclusion, our results indicate an effect of sex on OS in gastric/gastroesophageal cancer after neoadjuvant CTx, which might differ depending on the MSI status of the tumor and may also vary between patient cohorts treated with or without inclusion of a taxane compound in the chemotherapeutic regimen. Combined consideration of all these factors should be further evaluated in a larger number of patients, and this could contribute to more individualized treatment approaches for patients with gastric or gastroesophageal cancer.

## Supplementary Information

Below is the link to the electronic supplementary material.

Supplementary file1 (DOCX 174 KB) The data presented in this study are available in this article or supplementary material. 

## Data Availability

The data presented in this study are available in this article or supplementary material.
