# Supplementary material for: Microsatellite instability and sex-specific differences of survival in gastric cancer after neoadjuvant chemotherapy without and with taxane: An observational study in real world patients
Source: J Cancer Res Clin Oncol. 2023 Mar 31;149(10):7651–62. doi: 10.1007/s00432-023-04691-5 (PMC10374811; doi:10.1007/s00432-023-04691-5)
Supplement: Supplementary file 1 — Supplementary file1 (DOCX 174 KB) The data presented in this study are available in this article or supplementary material. [file 432_2023_4691_MOESM1_ESM.docx]

**Supplementary Material**

Supplementary **Table S1- S2**

Supplementary **Fig. S1** **– S2**

**Table S1** Chemotherapy regimens

| **Neoadjuvant chemotherapy** | **n %** | |
| --- | --- | --- |
| **Total** | **505** | **100** |
| **No Taxane** | **411** | **81.4** |
| Cis + 5-FU or Cap | 222 | 44.0 |
| Ox + 5-FU or Cap | 78 | 15.4 |
| Cis or Ox + 5FU or Cap + Epi | 102 | 20.2 |
| Cis or Ox + Ab | 9 | 1.8 |
| **Taxane included** | **94** | **18.6** |
| Cis+5FU + Doc or Pac  Ox + 5FU + Doc (FLOT)  FLOT + Ab | 29  62  3 | 5.7  12.3  <1 |

Cis, cisplatin; Ox, oxaliplatin; 5-FU, 5-fluorouracil; Cap, capecitabine;

Epi, epirubicin; Ab, antibody; Doc, doxetacel; Pac, paclitaxel

**Table S2** Association of MSI status with clinicopathological factors in patients treated with or without taxanes

| Variable | p values^a^ | |
| --- | --- | --- |
|  | platinum/fluoropyrimidine CTx | |
|  | without taxane | with taxane |
| Sex | 0.146 | 0.683 |
| Age | **0.044^b^** | 0.286^b^ |
| Laurén (intestinal vs. non-intestinal | 0.732 | 0.503 |
| Localisation (proximal vs. non-proximal | 0.159 | **0.008** |
| ypT (ypT0,1,2 vs. 3,4) | 0.477 | 0.091 |
| ypN (negative vs. positive) | 0.360 | 0.310 |
| M status (negative vs. positive) | 0.316 | 0.697 |
| R status ( R0 vs.R1) | 0.695 | 0.448 |
| Response (TRG1 vs. TRG2,3) | 1.000 | 0.167 |

^a^ Chi-squared or Fisher's exact tests, ^b^ Mann-Whitney U Test


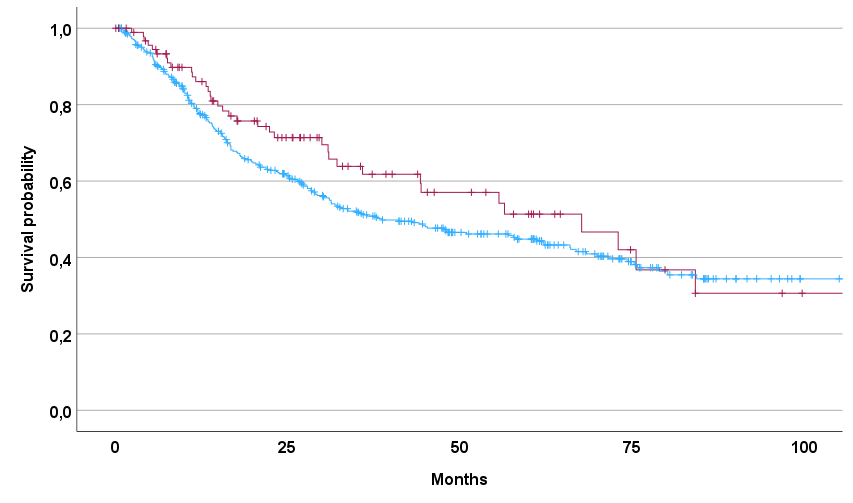


Number at risk

with taxane 94 46 22 8 2

without taxane 411 210 115 50 14

with taxane

without taxane

**Fig. S1** Discrimination of patients’ survival by CTx with and without taxane

Kaplan–Meier curves of patients treated with platinum/fluoropyrimidine neoadjuvant CTx with and without taxane are shown; p= 0.179; p of univariable Cox-regression.


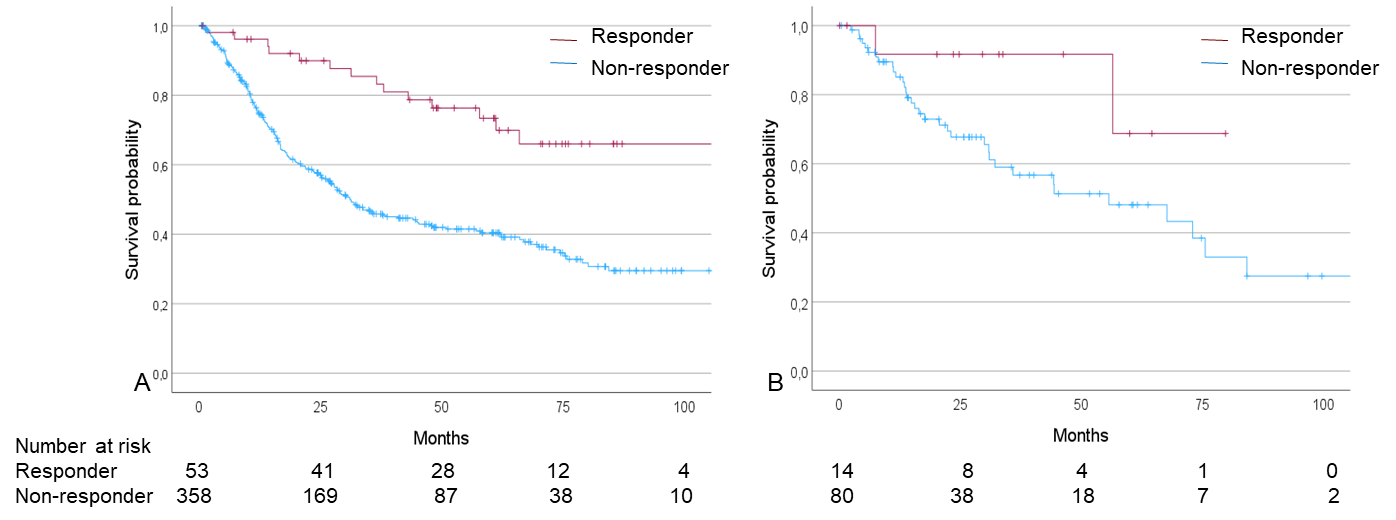


**Fig. S2** Discrimination of patients’ survival by response to CTx

Kaplan–Meier curves of responding (TRG1) and non responding (TRG2,3) patients are shown. Patients treated with neoadjuvant CTx A) without taxane, p<0.001; B) with taxane, p=0.105; p of univariable Cox-regression.
